# Passing Events and Topics

**Published:** 1920-11-13

**Authors:** 


### Passing Events and Topics.

### Churches and the Red Cross.

The heads of seventeen sects have recommended to their places of worship that on Armistice Sunday, November 14, a collection shall be taken on behalf of the work of the Joint Council of the British Red Cross Society and the Order of St. John.

### Salford Royal Hospital.

As a result of a house-to-house collection on behalf of the Royal Hospital, Salford, a sum of £6,000 has been received.

### Massage by Blind Soldiers.

The blind soldiers trained at St. Dunstan's in massage have opened a clinic at 18 Christopher Street, Finsbury Square, for the convenience of business men requiring treatment. Treatment will not be given unless prescribed by a doctor.

